# Trilaciclib use in extensive-stage small cell lung cancer (ES-SCLC): are clinical benefits seen in the real-world setting?

**DOI:** 10.1007/s00520-024-08828-1

**Published:** 2024-08-31

**Authors:** Joseph Elijah, Prantesh Jain, Allison Holdsworth, Jeffrey Baron, Eugene Przespolewski, Katy Wang, Kristopher Attwood, Christina Billias, Grace K. Dy

**Affiliations:** 1 Department of Pharmacy, Massachusetts General Hospital, Boston, MA USA; 2 School of Pharmacy and Pharmaceutical Sciences, Northeastern University, 140 The Fenway, Room 220, Boston, MA USA; 3 Department of Medical Oncology, Roswell Park Comprehensive Cancer Center, Buffalo, NY USA; 4 University at Buffalo School of Pharmacy and Pharmaceutical Sciences, Buffalo, NY USA; 5 Department of Pharmacy, Roswell Park Comprehensive Cancer Center, Buffalo, NY USA; 6 Department of Biostatistics and Bioinformatics, Roswell Park Comprehensive Cancer Center, Buffalo, NY USA

**Keywords:** ES-SCLC, Trilaciclib, Neutropenia, Carboplatin, Etoposide, Atezolizumab

## Abstract

**Background:**

Trilaciclib, in comparison to placebo plus carboplatin, etoposide, ± atezolizumab (PEA), has shown significant reductions in incidence of severe neutropenia (SN) among patients with extensive-stage small cell lung cancer (ES-SCLC). Despite these findings, real-world utility remains limited.

**Methods:**

A single-center quasi-experimental study compared trilaciclib + PEA (PEAT) versus PEA in ES-SCLC patients. The study period ranged from April 1, 2021 to July 31, 2022, for the PEAT recipients and February 1, 2020, to February 28, 2021, for PEA recipients. The primary endpoint evaluated was incidence of SN after cycle 1 and during the treatment period. Secondary endpoints included measures related to myelopreservation and patient outcomes.

**Results:**

Among 34 PEAT and 44 PEA patients, baseline characteristics were similar, except for a higher median age (69 vs 64 years) and more males (64.7% vs 38.6%) in the PEAT cohort. The PEAT cohort exhibited a lower SN rate (3%) versus the PEA cohort (18%), with statistical significance demonstrated on multivariate analysis (*p* = 0.015). Additionally, the PEAT cohort also demonstrated significant reductions in red blood cell transfusion requirements (3% vs 23%; *p* = 0.02), grade 3–4 anemia (6% vs 25%; *p* = 0.03), and grade 3–4 thrombocytopenia (0% vs 11%, *p* = 0.045).

**Conclusion:**

Trilaciclib, in combination with PEA, demonstrated an improvement in the safety profile without compromising survival outcomes in ES-SCLC patients. These findings underscore the potential benefits of incorporating trilaciclib in real-world clinical settings for enhanced patient care.

## Introduction

Extensive-stage small cell lung cancer (ES-SCLC) represents a therapeutic challenge due to the aggressive nature of tumor growth, early metastatic spread, and the lack of targetable mutations [1]. Nonetheless, platinum-based cytotoxic chemotherapy has remained the backbone of first-line treatment in combination with either atezolizumab or durvalumab even in the current era of immunotherapy [2, 3]. These cytotoxic agents can damage hematopoietic stem and progenitor cells (HSPCs) in the bone marrow, thereby resulting in chemotherapy-induced myelosuppression (CIM) [4]. This sequela may lead to chemotherapy dose reductions and/or delays, which can minimize the intended antitumor effects [5].

Cytotoxicity to HSPCs is particularly concerning in the ES-SCLC patient population because they are often elderly with multiple comorbid conditions and end-organ dysfunction, and thus the risk for CIM is magnified [6, 7]. The cytotoxic therapies utilized in SCLC are recognized in the 2022 Hematopoietic Growth Factors Guideline from the National Comprehensive Cancer Network (NCCN), as being associated with an intermediate to high risk for febrile neutropenia [8]. The myelosuppressive effects of chemotherapy are routinely managed with growth colony stimulating factors (GCSFs), erythrocyte stimulating agents (ESAs), red blood cell (RBC), and/or platelet transfusions [4]. With the exception of prophylactic G-CSF to ameliorate risk of neutropenic fever, these interventions are lineage-specific and administered after chemotherapy has inflicted myelotoxicity and thus are reactive as opposed to protective. Therefore, a tri-lineage myeloprotective intervention is warranted due to the well-recognized association of severe neutropenia (SN) and duration of neutropenia as risk factors for systemic infections, aside from mitigation of known risks of anemia and thrombocytopenia that can affect quality of life [9].

Trilaciclib is a selective, reversible cyclin-dependent kinase 4 and 6 (CDK4/6) inhibitor that takes advantage of the reliance of HSPCs on CDK4/6 activity for myeloproliferation [10–12]. CDK4/6 inhibition results in cell cycle arrest in the G1 phase. Intermittent administration of a transient CDK4/6 inhibitor such as trilaciclib prior to chemotherapy serves to protect HSPCs from chemotherapy-induced damage [12].

The SCLC population was specifically selected to investigate the role of trilaciclib because SCLC tumors replicate independently of CDK4/6 due to inactivation of the downstream retinoblastoma (RB) protein signaling arising from high prevalence of RB loss and/or mutation; thereby, assessment of the effect on the host is feasible and devoid of potential direct effects on the tumor [11, 12]. Two published phase I and/or II trials in patients with ES-SCLC who received trilaciclib versus those who did not prior to administration of carboplatin, etoposide + / − atezolizumab (PEA), demonstrated statistically significant improvements in the primary endpoints: duration of severe neutropenia in cycle 1 and incidence of severe neutropenia throughout the treatment period [13, 14]. There were also significant reductions in the severity of hematologic adverse effects, but the reductions in transfusions or use of ESA or GCSF were not consistently significant in statistical analyses. Moreover, there was no beneficial outcome seen in terms of PFS and OS although overall quality-of-life measures generally favored the trilaciclib group in exploratory analyses. Given the established use of G-CSF to overcome neutropenia-related complications, the aim of this analysis is to assess the utility of trilaciclib as well as the degree of patient benefit in the real-world setting regarding additional supportive care measures administered and survival outcomes to justify incorporation of trilaciclib into routine clinical practice.

## Methods

### Hospital

This retrospective, cohort study was conducted at Roswell Park Comprehensive Cancer Center in Buffalo, NY. Approval was granted by the Institutional Review Board (IRB), and research was conducted in accordance with the principles outlined in the Declaration of Helsinki.

### Study design

Adult patients (age ≥ 18 years) with confirmed ES-SCLC who received trilaciclib (PEAT) versus those who did not receive trilaciclib (PEA) prior to chemotherapy regimens containing PEA on days one through three of a 21-day cycle for up to six cycles were identified via the electronic health record (EHR) and included in the study. Two EHR reports were generated for this natural quasi-experimental approach to compare cohorts prior to and after FDA approval and commercial availability of trilaciclib. The first report included all ES-SCLC patients who received PEAT from April 1, 2021, to July 31, 2022, whereas the second report included ES-SCLC patients who received PEA from February 1, 2020, to February 28, 2021 (timeframe prior to adoption of trilaciclib into the institute’s drug formulary).

Patients were excluded if they received treatment only for limited-stage SCLC, trilaciclib was initiated with second or later cycles of PEA, there is inconsistent trilaciclib utilization, carboplatin dose AUC < 3.5 with cycle 1, primary prophylaxis with GCSF during cycle 1, active enrollment in a clinical trial and/or receipt of investigational drugs, they were lost to follow-up, and they are pregnant or lactating women. Study data obtained from the EHR included baseline (defined as within 30 days prior to initiation of cycle 1 PEA ± trilaciclib) patient demographic information and disease characteristics (age, gender, race, ECOG PS, lactate dehydrogenase (LDH), presence of brain metastases, smoking history, PD-L1 status, eGFR, carboplatin and etoposide dose at cycle 1, hemoglobin, platelets, absolute neutrophil count, lymphocytes, as well as receipt of chemotherapy prior to PEA for other malignant diseases).

The primary endpoints for the study were incidence of SN, defined as an absolute neutrophil count (ANC) of less than 0.5 cells × 10^9^ cell/L, after cycle 1 and throughout treatment duration. Secondary endpoints assessed were incidence of febrile neutropenia (FN), lineage-specific transfusion and/or growth-factor requirements (RBC, platelets, ESA, and GCSF) and total all-cause chemotherapy reductions and treatment delays during each cycle, IV antibiotic use, incidence of systemic infections, hospitalizations secondary to either systemic infections or febrile neutropenia as defined in the 2022 NCCN Prevention and Treatment of Cancer-Related Infections Guidelines, PFS, OS, and incidence of hematologic toxicities (anemia, thrombocytopenia, neutropenia, and lymphopenia) during each cycle by grade as defined in the Common Terminology Criteria for Adverse Events (CTCAE), version 5 [15, 16]. All the aforementioned data points were collected and stored in the REDCap web-based application [17, 18].

### Statistical analysis

The statistical analysis utilized in this study was modeled similarly to the one published in the trial by Daniel et al. which led to FDA approval for use prior to PEA in patients with ES-SCLC [12]. Incidence of SN was compared between the PEAT and the PEA cohort by using a one-sided Fisher’s exact test. The required sample size was calculated to be a minimum of 34 patients in both the PEAT and the PEA cohorts to have 90% power at a one-sided alpha of 0.025 to detect an absolute decrease of 34% in the incidence of severe neutropenia.

Demographic data was analyzed by using descriptive statistics (counts, percentages, means, and standard deviations). In the univariate analyses, categorical variables were compared using a one-sided Fisher’s exact test, and continuous variables were compared using the Mann–Whitney *U* test. PFS and OS were evaluated by the Kaplan–Meier estimate, with comparisons made using the log-rank test. Multivariate logistic or Cox regression was utilized for the pertinent primary and secondary endpoints and adjusted for the a priori selected variables age and sex. Odds and hazard ratios, with corresponding 95% confidence intervals, were obtained from model estimates. All analyses were conducted in the SAS v9.4 (Cary, NC) at a significance level of 0.05.

## Results

### Patient population

**Fig. 1:**
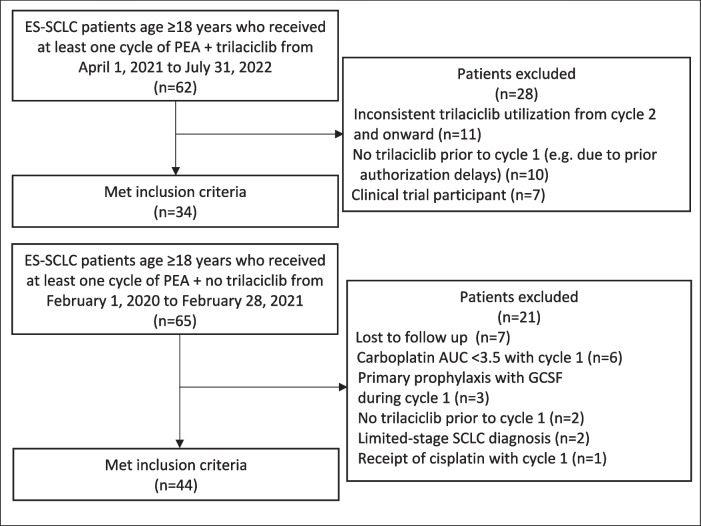
CONSORT diagram

Of the 62 patients identified who received PEAT, 34 met the inclusion criteria for review (Fig. 1). Inconsistent trilaciclib utilization from cycle two and onward was the most common reason for exclusion (*n* = 11). Likewise, 44 patients in the PEA cohort were included for review from an initial list of 65 patients. Lost to follow-up (*n* = 7), carboplatin AUC < 3.5 with cycle 1 (*n* = 6), and primary prophylaxis with GCSF during cycle 1 (*n* = 3) were the most common reasons for exclusion from the PEA cohort.

### Univariate analysis

**Fig. 2:**
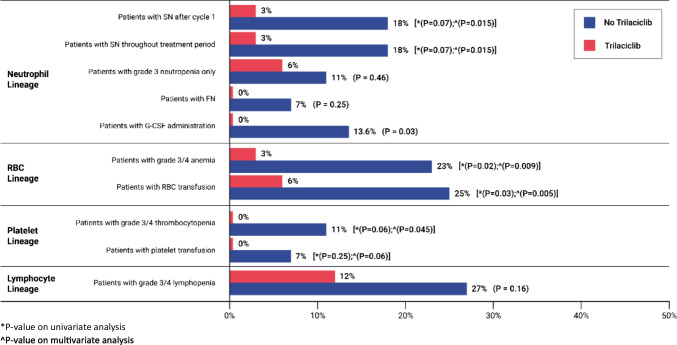
Summary of myelosuppression endpoints

**Fig. 3:**
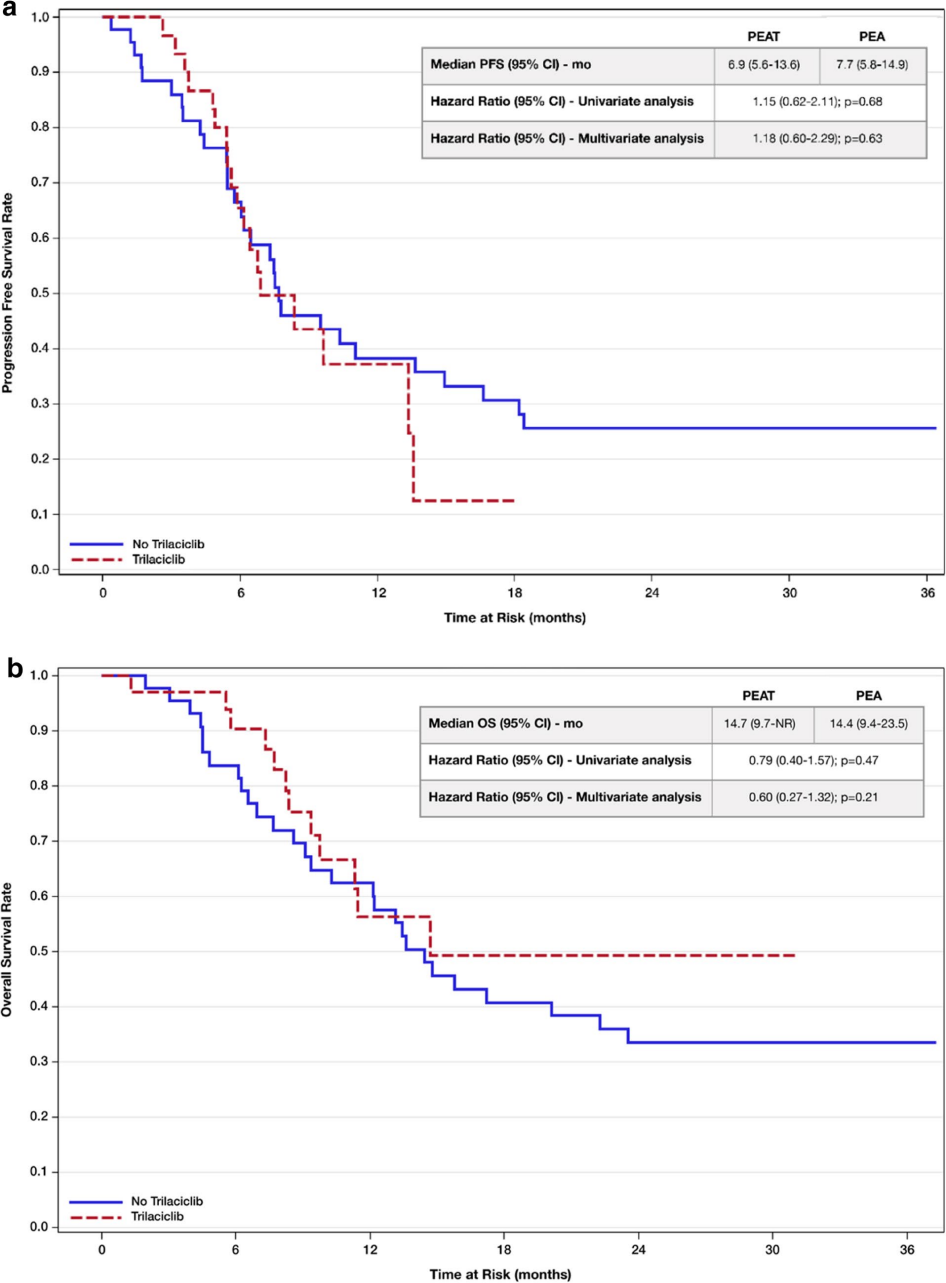
**a** Unadjusted Kaplan–Meier PFS rate estimate, **b** Unadjusted Kaplan–Meier OS rate estimate

Demographic and baseline clinical characteristics were similar between both cohorts except for older median age of patients (69 years old vs 64 years old) and higher proportion of male patients (64.7% vs 38.6%) in the PEAT cohort (Table 1). The PEAT cohort, when compared to the PEA cohort, demonstrated a numerically lower incidence of severe neutropenia after cycle 1 (3% vs 18%; *p* = 0.07), overall severe neutropenia (3% vs 18%; *p* = 0.07), grade 3 neutropenia (6% vs 11%; *p* = 0.46), and febrile neutropenia (0% vs 7%; *p* = 0.25). However, statistical significance was not met for all the neutrophil-related endpoints (Fig. 2). Likewise, platelet transfusion requirements as well as grade 3/4 thrombocytopenia and lymphopenia were not statistically different. GCSF administration was significantly higher in the PEA cohort (13.6% vs 0%; *p* = 0.03). Although the median number of packed red blood cell (PRBC) or platelet administration was lower in the PEAT cohort compared to PEA, this was not statistically significant (Table 2). ESA was not administered to either cohort for management of chemotherapy-induced anemia. Incidence of total all-cause chemotherapy reductions (*p* = 0.16), treatment delays (*p* = 0.44), systemic infections (*p* = 0.13), IV antibiotic administration (*p* = 0.06), and hospitalization secondary to infection or febrile neutropenia (*p* = 0.06) were also not statistically different (Table 3). However, the PEAT as compared to PEA cohort experienced a statistically significant reduction in number of patients with RBC transfusion requirements (3% vs 23%; *p* = 0.02) and grade 3/4 anemia (6% vs 25%; *p* = 0.03) (Fig. 2). PFS and OS between the two cohorts were not statistically different (Fig. 3).

**Table 1:** Baseline demographic and clinical characteristics

Characteristic, *n* (%)	PEAT (*N* = 34)	PEA (*N* = 44)
Age (years) at therapy initiation, median (range)	69 (55–85)	64 (49–84)
Gender		
Male	22 (64.7)	17 (38.6)
Female	12 (35.3)	27 (61.4)
Race		
White	33 (97.1)	38 (86.4)
Black	1 (2.9)	1 (2.3)
Other	0	5 (11.4)
Baseline LDH (g/dL)		
> ULN (618)	14 (41.2)	19 (43.2)
Within normal limits	17 (50.0)	18 (40.9)
Missing	3 (8.8)	7 (15.9)
Baseline ECOG PS		
0	6 (17.6)	10 (22.7)
1	18 (52.9)	25 (56.8)
2	10 (29.4)	9 (20.5)
Baseline eGFR (mL/min)		
< 30	1 (2.9)	0
30–59	5 (14.7)	4 (9.1)
≥ 60	28 (82.4)	40 (90.9)
Presence of brain metastases	14 (41.2)	16 (36.4)
Smoking history		
Current smoker	13 (38.2)	18 (41.0)
Former smoker	21 (61.8)	24 (54.5)
Never smoked	0	2 (4.5)
PD-L1 status		
Positive (≥ 1%)	4 (11.8)	4 (9.1)
Negative (< 1%)	2 (5.9)	5 (11.4)
Missing	28 (82.4)	35 (79.5)
History of chemotherapy prior to PEA*	8 (23.5)	8 (18.2)
Carboplatin AUC (mg/mL × min) dose at cycle 1, median (range)	5.0 (3.5–5.0)	5.0 (4.0–5.0)
Etoposide dose at cycle 1 (mg/m^2^), median (range)	100 (50–100)	100 (50–100)
Number of cycles, median (range)	4 (1–6)	4 (1–6)
Baseline hemoglobin (g/dL), median (range)	13.4 (10.3–16.8)	12.5 (7.8–15.7)
Baseline platelets (g/dL), median (range)	257.5 (145.0–680.0)	276.0 (75.0–711.0)
Baseline neutrophils (cells/µL), median (range)	5.8 (2.5–17.7)	6.3 (1.8–22.7)
Baseline lymphocytes (g/dL), median (range)	1.3 (0.4–3.0)	1.2 (0.4–4.9)

*Chemotherapy agents received consisted primarily of the following drug classes: platinum agents, topoisomerase inhibitors, taxanes, or antimetabolites

**Table 2:** Summary of supportive care product administration

Blood product, *n*	PEAT (*N* = 34)	PEA (*N* = 44)	*p* value
Total packed red blood cell (units), median (range)	1.0 (1.0–1.0)	2.5 (1.0–7.0)	0.33
Total platelets (units), median (range)	0	1 (1.0–4.0)	–-

**Table 3:** Summary of treatment-related delays and infection characteristics

Characteristic, *n* (%)	PEAT (*N* = 34)	PEA (*N* = 44)	*p* value
Total all-cause chemotherapy reductions, median (range)	0 (0–2)	0 (0–4)	0.16
Total treatment delay occurrences, median (range)	1 (0–3)	1 (0–2)	0.44
Diagnosis of systemic infections	0	4 (9.1)	0.13
Required IV antibiotic administration throughout treatment	0	5 (11.4)	0.06
Required hospitalization secondary to systemic infection or febrile neutropenia	0	5 (11.4)	0.06

### Multivariate analysis

The pertinent lineage-specific endpoints and survival outcomes were adjusted for difference in age and gender between the PEAT and PEA cohorts (Figs. 2 and 3). No statistically significant differences were seen between both cohorts regarding platelet transfusion requirements (*p* = 0.25), PFS (*p* = 0.63), and OS (*p* = 0.21). Likewise, incidence of grade 3/4 anemia (*p* = 0.009) and RBC transfusion requirements (*p* = 0.005) remained significantly lower in the PEAT versus the PEA cohort. However, a statistically significant difference was demonstrated in severe neutropenia after cycle 1 (*p* = 0.015), severe neutropenia throughout the treatment period (*p* = 0.015), and grade 3/4 thrombocytopenia (*p* = 0.045) between the PEAT and PEA cohorts.

## Discussion

Trilaciclib administration prior to PEA demonstrated a significant reduction in the incidence of SN throughout the treatment period in comparison to placebo when evaluated in the randomized phase II trial by Daniel et al., which led to FDA approval for utilization prior to PEA in patients with ES-SCLC [14]. The univariate analysis in our real-world study demonstrated a numerically lower but not a statistically significant reduction in the incidence of SN after cycle one and throughout the treatment period. However, patients belonging to the PEAT cohort in our study were older in age (median age of 69 years old vs 64 years old) and were disproportionately made up of male patients (65% vs 39%) when compared to the PEA cohort. Baseline patient demographic and clinical characteristics were well balanced between both cohorts in the phase II trial, which may be why the incidence of SN after cycle one and throughout treatment became statistically significant when correcting for the difference in age and gender between the cohorts in our study. Therefore, it is reassuring that real-world ES-SCLC patients who received trilaciclib prior to PEA for at least four cycles (four cycles was the median for both cohorts in this study and max number of cycles administered in the phase II trial) experienced a significant reduction in incidence of SN throughout the treatment cycle.

The GIT28-05 randomized phase II PEAT vs PEA trial did not demonstrate a significant reduction in the incidence of lineage-specific transfusion requirements, grade 3/4 anemia and thrombocytopenia, FN, as well as GCSF administration [14]. In contrast, the incidence of grade 3/4 anemia and thrombocytopenia, RBC transfusion requirements, and GCSF administration were significantly lower in the PEAT when compared to the PEA cohort in our study. Our analysis included patients who received prior chemotherapy (e.g., platinum-based therapy for limited-stage or ES-SCLC) in PEAT (23.5%) and PEA (18.2%) cohorts, whereas such patients were excluded in the GIT28-05 study. Although participants in the GIT28-05 presumably had better bone marrow reserve compared to our patient population and received a maximum of four cycles only, higher frequency of patients with grade 3/4 anemia and RBC transfusion needs was encountered in the PEAT treatment arm of the GIT28-05 trial relative to the PEAT cohort at our center, while RBC-related events observed in the GIT28-05 control group were similar to our PEA only cohort. Thus, while numerically in favor of PEAT, the magnitude of benefit in terms of RBC-related endpoints was not statistically significant in the GIT28-05 study. Nonetheless, two pooled analyses of three randomized phase II trials for SCLC (two in the platinum-based chemotherapy combination setting, one in the topotecan-based treatment setting) demonstrated significantly lower grade 3/4 anemia as well as RBC transfusion requirements in patients who received trilaciclib, which likely contributed to improved patient-reported outcome measures (e.g., fatigue, physical, and functional well-being) [19, 20]. Furthermore, the RBC and platelet sparing effects of trilaciclib are desirable in order to allocate the relevant replacement blood products to patients who require transfusions, which is of particular relevance during blood product shortages.

Infection-related outcomes were not significantly different between the PEAT and PEA cohorts. The PEAT group in comparison to the PEA group demonstrated a numerically lower incidence of systemic infections, requirement of IV antibiotic administration throughout treatment, and hospitalization secondary to systemic infection or FN. A similar result for the aforementioned outcomes was demonstrated in the GIT28-05 [14] and in the pooled analyses as well [19, 20]. The PEAT versus the PEA cohort in our study did not demonstrate a significant difference in PFS (6.9 vs 7.7 months; *p* = 0.68) and OS (14.7 vs 14.4 months; *p* = 0.47), recapitulating the observed survival outcomes in the GIT28-05 phase II studies [13, 14]. The PFS and OS outcomes in our study were also similar to what was observed in the pivotal IMPower133 trial and thus reassure that trilaciclib utilization does not produce detrimental effects on survival outcomes [2].

The strengths of this study include the natural quasi-experimental study design and the assessment of primary and secondary endpoints originally evaluated in the pivotal phase II trilaciclib trial. Limitations of this study are the retrospective and single-center nature, lack of quality-of-life patient-reported outcomes (PRO) due to variability in EHR documentation amongst providers as well as lack of standardized system-wide uniform collection of PROs. A lower threshold for RBC transfusion (hemoglobin of 7 g/dL as opposed to 8 g/dL) was also adopted by our institution in early 2022 due to a national blood product shortage. This restriction for blood product administration may potentially affect decisions on whether to administer PRBCs to patients who were included in our study. However, this would not have impacted the unbiased observation that the PEAT cohort had lower grade 3 or 4 anemia rates when compared to the PEA group, with the proportion of patients receiving PRBC administration reflected similarly in each group. Hence, the observed difference in transfusion rate is unlikely to be spurious.

## Conclusion

Trilaciclib maintained its previously observed effects with regard to reducing the incidence of SN. Real-world patients in this study exhibited significantly less grade 3/4 thrombocytopenia similar to what was seen in the GIT28-05 phase II randomized study. Rates of grade 3/4 anemia as well as RBC transfusion requirements were statistically in favor of the trilaciclib-treated group for our study, which did not reach statistical significance in the GIT28-05 study. Our study independently shows that trilaciclib administration prior to PEA for patients with ES-SCLC provides myeloprotection and does not negatively impact survival outcomes. Trilaciclib is also FDA approved for myeloprotection prior to administration of topotecan in patients with ES-SCLC [21]. Future studies validating its benefits in combination with lurbinectedin for ES-SCLC (NCT05578326) or in limited stage high-risk-SCLC patients undergoing chemoradiation (e.g., patients with reduced renal function) are likely to be of value as well.

## Data Availability

The study’s supporting data cannot be openly accessed for reasons of sensitivity, but can be made available upon request from the corresponding author.
